# Orphan Toxin OrtT (YdcX) of *Escherichia coli* Reduces Growth during the Stringent Response

**DOI:** 10.3390/toxins7020299

**Published:** 2015-01-29

**Authors:** Sabina Islam, Michael J. Benedik, Thomas K. Wood

**Affiliations:** 1 Department of Chemical Engineering, the Pennsylvania State University, University Park, PA 16802-4400, USA; E-Mail: sui112@psu.edu; 2 Department of Biology, Texas A&M University, College Station, TX 77843-3258, USA; E-Mail: benedik@tamu.edu; 3 Department of Biochemistry and Molecular Biology, the Pennsylvania State University, University Park, PA 16802-4400, USA

**Keywords:** toxin/antitoxin, orphan toxin, stringent response

## Abstract

Toxin/antitoxin (TA) systems are nearly universal in prokaryotes; toxins are paired with antitoxins which inactivate them until the toxins are utilized. Here we explore whether toxins may function alone; *i.e.*, whether a toxin which lacks a corresponding antitoxin (orphan toxin) is physiologically relevant. By focusing on a homologous protein of the membrane-damaging toxin GhoT of the *Escherichia coli* GhoT/GhoS type V TA system, we found that YdcX (renamed OrtT for orphan toxin related to tetrahydrofolate) is toxic but is not part of TA pair. OrtT is not inactivated by neighboring YdcY (which is demonstrated to be a protein), nor is it inactivated by antitoxin GhoS. Also, OrtT is not inactivated by small RNA upstream or downstream of *ortT*. Moreover, screening a genomic library did not identify an antitoxin partner for OrtT. OrtT is a protein and its toxicity stems from membrane damage as evidenced by transmission electron microscopy and cell lysis. Furthermore, OrtT reduces cell growth and metabolism in the presence of both antimicrobials trimethoprim and sulfamethoxazole; these antimicrobials induce the stringent response by inhibiting tetrahydrofolate synthesis. Therefore, we demonstrate that OrtT acts as an independent toxin to reduce growth during stress related to amino acid and DNA synthesis.

## 1. Introduction

Folic acid (vitamin B_9_) plays a pivotal role in both prokaryotic and eukaryotic cells for the biosynthesis of many cellular components [1]. Humans cannot synthesize folates and so depend on an exogenous supply, whereas most prokaryotes synthesize this essential cofactor *de novo* [2] which makes the folate metabolic pathway an interesting target in urinary tract infections [3], chemotherapy, and malarial disease treatment [4]. The reduced form of folic acid, tetrahydrofolate (THF) serves as the donor of one-carbon units in myriad metabolic pathways involved in the formation of purines, thymidine, glycine, and methionine [5]. THF is also required for synthesizing formylmethionyl-tRNA^fMet^ which is essential for initiation of protein synthesis in bacteria [6]. Depletion of the intracellular THF pool causes “thymineless death” in rich medium where bacteria undergo cell-death due to thymine starvation or leads to a stringent response in minimal medium where the bacterial population enters a non-dividing state due to amino acid starvation [5]. The stringent response involves accumulation of the regulatory alarmone guanosine tetraphosphate (ppGpp) [7]. One of the ways to cope with nutritional stress for bacteria is activation of toxin-antitoxin (TA) systems through ppGpp to achieve a non-metabolizing dormancy state [8,9]. This quiescent state is called “persistence” in which bacterial cells are tolerant to antibiotics.

TA systems are diverse and abundant in free-living prokaryotes [10]. A TA system usually consists of two genes in an operon that encode for a stable toxin that damages the bacterial cell that makes the toxin (rather than affecting another cell) by inhibiting a critical physiological step and a labile antitoxin, which protects the host by blocking the deleterious activity of the toxin [11]. The cellular targets of these toxins are quite diverse including DNA replication machinery, mRNA, protein synthesis, cell membranes, ATP synthesis [12], and DNA [13]. To date, five types of TA systems have been categorized depending on the role of the antitoxins. In type I systems, antitoxin RNA silences the toxin by binding to the toxin mRNA [14], and in type III systems, the antitoxin RNA binds to the toxin via its organized repeat motifs [15]. In type II, IV, and V systems, the protein antitoxin either directly binds the toxin (type II) [16], interferes with the binding of the toxin to its target (type IV) [17], or degrades the toxin mRNA via its specific enzymatic endoribonuclease activity (type V) [18]. The recently characterized type V TA system comprises of a membrane lytic toxin, GhoT and an unique antitoxin, GhoS which is not labile during stress and does not participate in transcriptional control of the TA pair; rather, it functions as an antitoxin by specifically cleaving *ghoT* mRNA.

Although initially discovered related to plasmid maintenance [19], their ubiquitousness in bacterial chromosomes have made TA loci the subject of intense scrutiny to unveil their enigmatic role in cell physiology. In most cases, deletion of a single TA pair rarely has any impact on cell physiology [20] and ectopic expression of toxins is often required to study their effect on bacterial growth. For example, persistence was unchanged with deletion of single toxin genes even with severe antibiotic stress [21] whereas successive deletion of 10 type II TA systems led to reduced persistence level [20].

Despite these difficulties in determining physiological roles, TA systems are clearly phage inhibition systems [15,22,23] and stress-response elements [24,25]. There is also increasing evidence supporting the role of TA systems in achieving bacterial persistence [26,27,28]. For instance, deletion of type II toxin gene *mqsR* and the TA locus *mqsRA* both reduced persistence level [27]. Supporting this result, type I toxin TisB was directly linked with persistence as absence of *tisAB/istR* locus led to significant reduction in ciprofloxacin induced persistence [28]. Furthermore, expression of TA modules (RelBE, MazEF, DinJYafQ, MqsR, YoeB) were induced in a transcriptome study performed with isolated *E. coli* persister cells [29].

Although the classical paradigm for TA systems involves adjacent co-transcribed genes encoding a toxin and its specific antitoxin, several studies have described exceptions to this model such as cross interactions between non-cognate pairs [10,30,31]. Note that these cross interactions are distinct from TA cascades in which one TA system regulates another as shown with MqsR/MqsA regulation of GhoT/GhoS [32]. Another exception to the general TA concept is the extrinsic regulation of toxins by antitoxins encoded *in trans*. For example, the chromosomally encoded *ccd_Ech_* antitoxin was shown to act on a plasmid-encoded toxin *ccd_F_* to protect the cells from postsegregational killing [33]. Furthermore, MazF-mediated toxicity was inhibited by the remotely-encoded antitoxin MrpC in *Myxococcus xanthus* [34]. In addition to these exceptions, toxic endolysin genes are often found in cryptic prophage remnants such as PhyL in *Bacillus anthracis* [35] and LysA in *Streptococcus gordonii* [36] without the antitoxin genes.

Along with the consistent high specificity of nearly all toxins for their adjacent antitoxins, all but one of the characterized TA systems consists of two-components with the exception of the three-component system ω-ε-ζ of *Streptococcus pyogenes* [37]. Here, we demonstrate the importance of a one-component system by characterizing a new toxin, YdcX (renamed OrtT for orphan toxin related to tetrahydrofolate) in the chromosome of the model organism *E. coli* K12. We demonstrate that this new toxin is similar to GhoT of the type V GhoST TA system, but it lacks an antitoxin and is not regulated by GhoS antitoxin; hence, it is an orphan toxin. We also determine that OrtT functions as a toxin by damaging the cell membrane and reducing intracellular ATP level and that OrtT has a distinct physiological role, which is to reduce cellular growth and metabolism in response to the stringent response induced by depletion of THF.

## 2. Results

### 2.1. OrtT is a Proteic Toxin that Increases Persistence

**Figure 1:**
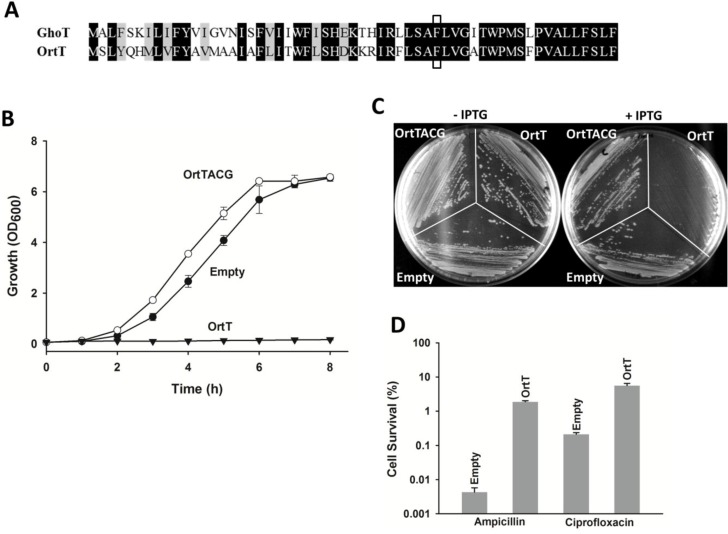
OrtT is a proteic toxin that increases persistence. (**A**) Sequence homology between GhoT and OrtT is shown, and the conserved Phe38 residue is boxed; (**B**) Growth of BW25113 wild-type cells harboring different plasmids in LB medium with chloramphenicol (30 µg/mL) with 1 mM IPTG introduced at 1h. Two independent cultures were evaluated. Error bar represent standard deviation; (**C**) Growth of BW25113 wild-type cells harboring different plasmids on LB plates with chloramphenicol (30 µg/mL) with or without 1 mM IPTG. Two independent cultures were evaluated; (**D**) Persistence assay with BW25113 wild-type cells after 3 h ampicillin (100 µg/mL) or ciprofloxacin (5 µg/mL) treatment with or without OrtT production. Two independent cultures were evaluated. Error bar represent standard deviation. Plasmid abbreviations: Empty, pCA24N; OrtT, pCA24N-*ortT*; OrtTACG, pCA24N-*ortT*ACG which lacks a start codon.

OrtT is the same size (57 aa) and shares 63% protein identity with toxin GhoT (Figure 1A). Hence, we investigated whether OrtT is a toxin. We found that production of OrtT from pCA24N-*ortT* caused severe growth inhibition (Figure 1B,C); hence, OrtT is a toxin. To determine if OrtT works as a protein toxin, the start codon was mutated from A*T*G (Met) to A*C*G (Thr) to generate pCA24N-*ortT*ACG. The wild-type strain harboring pCA24N-*ortT*ACG with inactivated protein OrtT was able to grow with full induction (Figure 1B). These results demonstrate that OrtT is a toxin that functions as a protein.

Ectopic expression of toxins such as MqsR increases persistence [27] since cells have reduced metabolism [38]. Hence, we tested if OrtT production influences persister cell formation by producing OrtT from pCA24N-*ortT*. As expected, persistence increased 430 fold with ampicillin treatment and 27 fold with ciprofloxacin treatment relative to cells with an empty vector (Figure 1D). Note that, generally persistence fold changes are lower by one to two orders of magnitude for ciprofloxacin treatment compared to ampicillin treatment [39] and that the induction conditions of *ortT* are different for the experiments in Figure 1B,D. Together, these results corroborate that OrtT is a bona-fide toxin that helps the cells to enter a dormant state.

### 2.2. OrtT Lyses Cells through Membrane Damage and Reduces ATP

**Figure 2:**
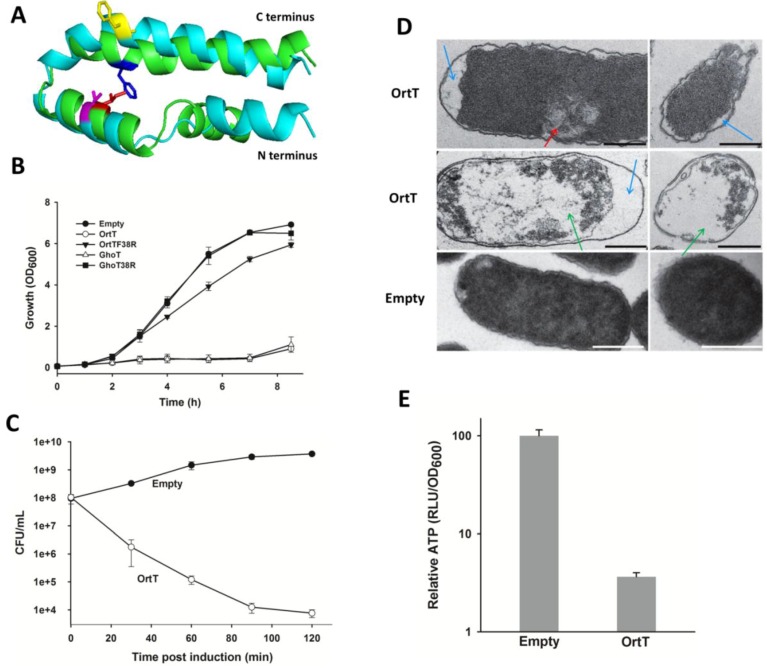
OrtT causes cell lysis via membrane damage and reduces cellular ATP. (**A**) Superimposed protein model of GhoT (green) and OrtT (cyan). Phe38 is blue for GhoT and yellow for OrtT, and Ile21 is red for GhoT and magenta for OrtT; (**B**) Growth of BW25113 wild-type cells carrying different plasmids in LB medium with chloramphenicol (30 µg/mL) with 0.1 mM IPTG introduced at 1h. Two independent cultures were evaluated. Error bar represents standard deviation; (**C**) Cell lysis assay with BW25113 wild-type cells producing OrtT or with empty vector. LB medium was used with chloramphenicol (30 µg/mL) and 2 mM IPTG (added at OD_600_ 0.5). Two independent cultures were evaluated. Error bar represents standard deviation; (**D**) TEM images of BW25113 wild-type cells producing OrtT (top two rows) or with empty vector (bottom row). Left column shows longitudinal sections, and the right column shows horizontal sections. The blue arrow shows the enlargement of the periplasmic space, the red arrow shows nucleoid condensation, and the green arrow shows the empty cytoplasmic space. Scale bar indicates 0.5 µm; (**E**) Change in relative intracellular ATP level due to OrtT production in BW25113 wild-type cells. LB medium was used with chloramphenicol (30 µg/mL) and cells were treated with 1 mM IPTG (added at OD_600_ 0.5) for 3 h. The ATP levels were calculated as RLU/OD_600_ (relative light units/optical cell density at 600 nm) and normalized to that of the strain with the empty vector pCA24N. Plasmid abbreviations: Empty, pCA24N; OrtT, pCA24N-*ortT*; OrtTF38R, pCA24N-*ortT*F38R having Phe38 substituted by Arg; GhoT, pCA24N-*ghoT*; GhoTF38R, pCA24N-*ghoT*F38R having similar F38R substitution.

Like GhoT, OrtT is predicted to be a hydrophobic inner-membrane protein with two transmembrane domains (residue 6 to 26 and 34 to 54) [40]. To investigate the structural resemblance of OrtT with GhoT, we modelled both proteins using PHYRE2 [41]. The modelled monomeric proteins have high structural identity as can be seen from the superimposed protein structures in Figure 2A. Previously, introduction of the F38R substitution abolished GhoT toxicity [42]; hence, since the Phe38 residue is conserved in both proteins (boxed nt, Figure 1A), we tested whether Phe38 bears the same functional-significance for OrtT toxin by constructing pCA24N-*ortT*F38R. As shown in Figure 2B, OrtT-mediated toxicity was reduced for OrtTF38R. These results suggest that OrtT is likely a membrane protein that functions in a manner similar to GhoT. Note that, we tried to visualize OrtTF38R via Western blot analysis but like GhoT [42], this toxic protein could not be detected (Figure S1).

Production of GhoT leads to cell lysis [18]. Hence, to characterize toxin OrtT, we tested if ectopic expression of *ortT* lyses cells and found that production of OrtT in wild-type cells caused 99.99% of the bacterial population to undergo cellular lysis within two hours after induction (Figure 2C). This suggests OrtT damages the cell membrane.

To confirm that OrtT causes membrane damage, we performed transmission electron microscopy (TEM) with wild-type cells producing toxin OrtT and compared them to wild-type cells with an empty vector. We found there was a dramatic change in cell morphology due to OrtT production; the two most common phenomena were (i) nucleoid condensation and enlargement of the periplasmic space; and (ii) cellular component leakage leading to almost a hollow cytoplasmic space (top two panels, Figure 2D). In contrast, cells with the empty vector looked healthy with an intact periplasm and cytoplasm.

Next, we tested whether OrtT-mediated membrane damage leads to loss of cellular ATP level by measuring intracellular ATP concentration. Production of OrtT caused a 27-fold loss in ATP level when compared with the empty vector strain (Figure 2E). This suggests that proton-motive force (PMF) is disrupted in OrtT producing cells. Collectively, these results corroborate that OrtT is most likely a membrane protein that leads to cell lysis via membrane damage and depletion of intracellular ATP pool.

### 2.3. OrtT is an Orphan Toxin

**Figure 3:**
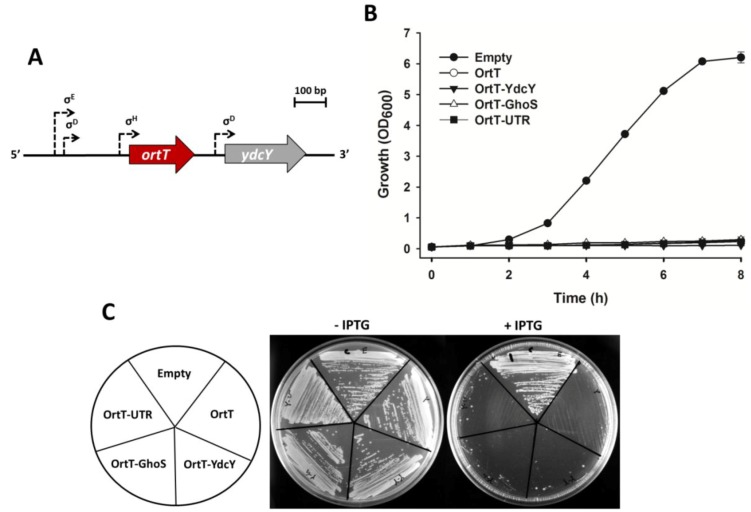
OrtT is an orphan toxin. (**A**) The chromosomal region around *ortT* gene in *E. coli*. *ortT* is represented by a red arrow and *ydcY* is shown by the grey arrow. Three predicted promoters (*i.e.*, σ^E^, σ^D^, and σ^H^) for *ortT* and one predicted promoter (*i.e.*, σ^D^) for *ydcY* are shown by dashed arrows; (**B**) Growth of BW25113 wild-type cells harboring empty plasmid, plasmid carrying *ortT* only or *ortT* with *ydcY*, *ghoS*, or the intervening region upstream and downstream of *ortT* (UTR) in LB medium with chloramphenicol (30 µg/mL) with 1 mM IPTG introduced at 1 h. Two independent cultures were evaluated. Error bar represents standard deviation; (**C**) Growth on LB plates with chloramphenicol (30 µg/mL) with or without 1 mM IPTG. Plasmid abbreviations: Empty, pCA24N; OrtT, pCA24N-*ortT*; OrtT-YdcY, pCA24N-*ortT*-*ydcY*; OrtT-GhoS, pCA24N-*ortT*-*ghoS*; OrtT-UTR, pCA24N-*ortT*-*UTR*. Two independent cultures were evaluated.

To further characterize this new toxin, we explored if it forms a toxin-antitoxin pair by interacting with other protein/RNA. Although *ortT-ydcY* does not form an operon (Figure 3A), we tested whether YdcY can act as an antitoxin by constructing pCA24N-*ortT-ydcY* (Figure S2A). Using this plasmid we found that YdcY does not act as an antitoxin as OrtT-mediated toxicity was not reduced when YdcY was co-produced with OrtT (Figure 3B). Note that YdcY was confirmed to be a protein by running an SDS-PAGE (Figure S3).

GhoS is the antitoxin for GhoT, and it functions by cleaving the *ghoT* transcript [18]. Since OrtT is similar to GhoT, we tested whether *ortT* mRNA is a substrate for GhoS by constructing pCA24N-*ortT-ghoS* (Figure S2B). We found that GhoS does not attenuate OrtT toxicity (Figure 3B). These plasmids (*i.e*., pCA24N-*ortT-ydcY* and pCA24N-*ortT-ghoS*) were constructed in a way that the toxin and putative antitoxin genes are under the control of the same promoter. Note that such co-expression of toxins and antitoxins from the same promoter has been used for different TA systems such as MqsRA (Figure S4) [43] and VapBC [44].

We also considered the possibility of OrtT to be regulated by an RNA antitoxin. Type I toxin genes are generally secluded by large intergenic distances ranging from 492 to 1181 bp upstream and 282–708 bp downstream [14]. Hence, it was unlikely for OrtT to be a member of type I TA family considering the relatively small intergenic distances (*i.e*., 194 bp upstream and 85 bp downstream). Nonetheless, we tested for RNA interactions by cloning the upstream and downstream intergenic region between *ortT* and neighboring genes *yncL* and *ydcY*, respectively, to construct pCA24N-*ortT-*UTR (Figure S2C,D). We found that RNA both upstream and downstream of *ortT* did not reduce its toxicity (Figure 3B). Note that previously such a construct has led us to identify the RNA antitoxin partner RalA for the toxin RalR [13].

To verify that an antitoxin for OrtT is not coded elsewhere within the genome, we performed an extensive search for potential antitoxins by constructing a genomic library by partially digesting BW25113 genomic DNA and cloning the DNA fragments downstream of *ortT* gene in the pCA24N-*ortT*-B construct. The library of cloned plasmids was then transformed into BW25113 cells and screened for an antitoxin by growing the transformants in presence of IPTG to induce *ortT* expression. With about 3-fold coverage of the genome and an average insert size of 622 bp, we found that the constructs that were able to survive OrtT toxin expression were those with defective plasmids (*i.e*., those lacking a functional *ortT* gene). Therefore, no antitoxin partner for OrtT was identified from these four sets of experiments which confirm that OrtT is an orphan toxin.

### 2.4. Physiological Relevance of Toxin OrtT

**Figure 4:**
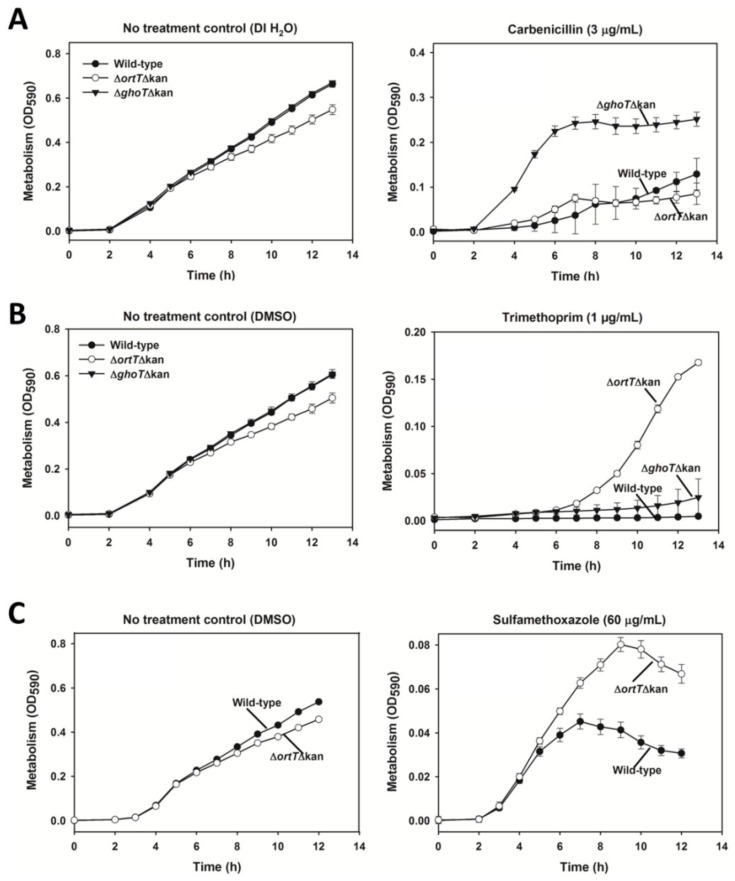
OrtT reduces cellular metabolism under THF-related antimicrobial stress. (**A**) Metabolic activity of the wild-type, *∆ortT ∆Kan*, and *∆ghoT ∆Kan* strains in response to 3 µg/mL carbenicillin or water (negative control); (**B**) Metabolic activity of the wild-type, ∆*ortT ∆Kan*, and *∆ghoT ∆Kan* strains in response to 1 µg/mL TMP or DMSO (negative control); (**C**) Metabolic activity of the wild-type strain and *∆ortT ∆Kan* in response to 60 µg/mL SMZ or DMSO (negative control). Two independent cultures were evaluated for each experiment. Error bar represents standard deviation.

Because both OrtT and GhoT are toxins that corrupt the membrane, we investigated if these two toxins serve a similar role in cell physiology. GhoT decreases cellular metabolism in the presence of carbenicillin and cefoxitin helping cells withstand this kind of membrane stress [42]. Hence, we first investigated whether OrtT was involved in the same pathway to slow metabolism in presence of carbenicillin. Surprisingly, OrtT was not involved in the stress response against carbenicillin (Figure 4A) since cells which lack OrtT (*i.e*., *∆ortT ∆Kan*) had low metabolism like wild-type cells whereas cells lacking GhoT (*i.e*., *∆ghoT ∆Kan*) showed high metabolism under this stressful condition. Note that only strains which lack the antibiotic resistance marker were used so that the only difference between the strains is deletion of the toxin gene.

Next, we investigated whether OrtT participates in a similar adaptive stress response with other antimicrobials. We took advantage of the PortEco database that indicates the results for large-scale phenotypic screening for all of the Keio mutants and that indicates *∆ortT* has a negative fitness score for trimethoprim (TMP) [45]. TMP is an antifolate drug that inhibits the synthesis of THF by binding to dihydrofolate reductase (DHFR) [46]. THF plays vital role in synthesis of amino acid serine and methionine (Figure S5), and low THF induces the stringent response and causes bacteria to enter into a non-proliferating state [5]. In addition, THF is an essential precursor for purine and thymidine triphosphate synthesis (Figure S5) and interference with THF metabolism inhibits bacterial DNA synthesis [47]. We found that the *∆ortT ∆Kan* mutant had dramatically higher metabolism compared to wild-type strain in response to 1 µg/mL TMP (Figure 4B), indicating OrtT acts as a metabolic brake to slow cellular metabolism under conditions that are unfavorable for amino acid and DNA synthesis. Critically, while the *∆ortT ∆Kan* strain was metabolically active in the presence of TMP, the *∆ghoT ∆Kan* strain was as sensitive to TMP as the wild-type cells (Figure 4B). These results indicate that toxins OrtT and GhoT have different physiological roles in cell fitness even though they share 63% protein identity.

Furthermore, to corroborate that OrtT acts specifically in the THF synthesis pathway, we performed a similar metabolic assay using sulfamethoxazole (SMZ). SMZ is often used along with TMP for their synergistic effect against bacteria since SMZ inhibits dihydropteroate synthetase, whereas TMP inhibits DHFR [48] (Figure S6). As expected, the *∆ortT ∆Kan* mutant showed higher metabolism compared to the wild-type host in response to 60 µg/mL SMZ (Figure 4C) which corroborates the previous result with TMP.

**Figure 5:**
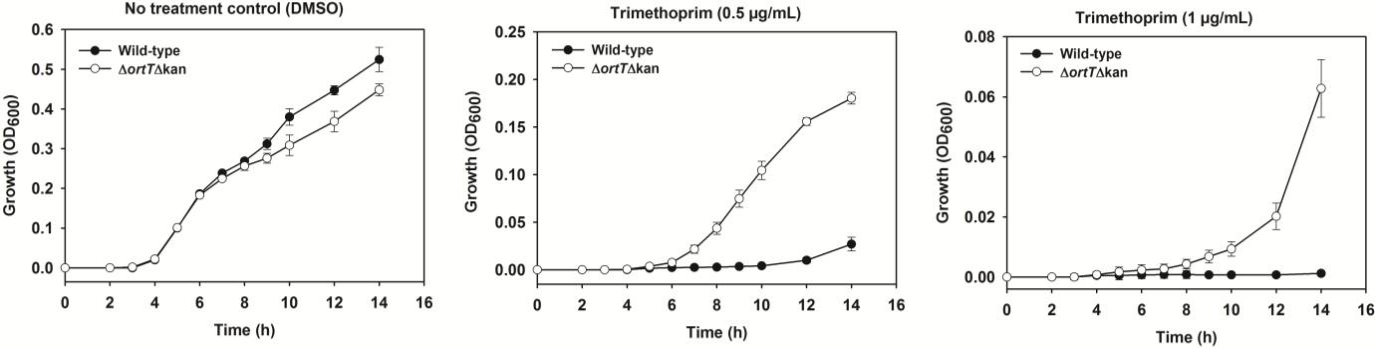
OrtT reduces cellular growth in the presence of TMP. Growth of the wild-type and *∆ortT ∆Kan* strains in response to 0.5 and 1 µg/mL TMP or DMSO (negative control). Two independent cultures were evaluated for each experiment. Error bar represents standard deviation.

Since metabolic activity is measured at 590 nm and cell growth is measured at 600 nm, we suspected OrtT might also influence cell growth when challenged with TMP. To investigate this, we performed the growth assay with wild-type and Δ*ortT ∆Kan* cells with or without TMP treatment. As expected, the mutant lacking *ortT* showed higher growth than that of the wild-type cells in the presence of TMP (Figure 5). Note that, the OD_600_ values for the growth experiment (Figure 5) are lower compared to the metabolic activity values at OD_590_ (Figure 4B) since the metabolic activity values include both growth and metabolism. Collectively, all these lines of evidence indicate that, OrtT is not a redundant GhoT-like toxin; rather, it is important for maintaining cell fitness during stress related to the stringent response.

### 2.5. Regulation of ortT

Since cellular growth and metabolism was higher in the absence of *ortT*, we reasoned that *ortT* is activated during the stringent response. To explore this possibility, we studied whether *ortT* expression is elevated under several conditions that should elevate ppGpp using quantitative real-time reverse-transcription PCR (qRT-PCR). Four different conditions were used to induce the stringent response: TMP [49], native RelA production [50], mupirocin (MUP) [51], and serine hydroxamate (SHX) [52]; for all four conditions, *ortT* expression was induced (Table 1 and Table S1). Among these, *ortT* expression was highest with SHX treatment and lowest with RelA production. The reason for such low expression of *ortT* in the case of RelA production could be that the activity of full-length native RelA protein is not constitutive and depends on amino-acid starvation condition [50].

To investigate the mechanism of *ortT* activation, we tested whether this toxin gene is expressed via the predicted sigma E and H-dependent promoters (Figure 3A) since sigma E (RpoE) is activated by ppGpp [53] and sigma H (RpoH) is under the regulation of sigma E [54]. We activated sigma E via heat-shock stress [55] and qRT-PCR showed that *ortT* was again activated (Table 1 and Table S1). Therefore, these results demonstrate that OrtT plays a vital role in the adaptive stress response of the cells since *ortT* is induced under nutrient limiting conditions most likely via transcription factor sigma E and H.

**Table 1:** Summary of qRT-PCR results to investigate whether *ortT* expression is induced under stringent conditions. All qRT-PCR experiments were performed with two biological replicates each with two technical replicates.

Conditions	Strains	Fold Change of *ortT* Induction	Treatment Description
TMP treatment	BW25113	3.1 ± 1.5	Exponentially-growing cells were treated with TMP (75 µg/mL) for 2 h.
RelA production	BW25113/pCA24N BW25113/pCA24N-*relA*	2.3 ± 0.6	Exponentially-growing cells were treated with IPTG (1 mM) to induce RelA production for 2 h.
MUP treatment	BW25113	4.61 ± 0.29	Exponentially-growing cells were treated with MUP (100 µg/mL) for 2 h.
SHX treatment	BW25113	89 ± 80	Exponentially-growing cells were treated with SHX (1 mg/mL) for 2 h.
Heat shock	BW25113	2.0 ± 0.2	Exponentially-growing cells were shifted from 30 °C to 43 °C for 30 min.

## 3. Discussion

In our previous study, the physiological role of type V toxin GhoT was ascertained; GhoT causes membrane damage by reducing cellular ATP level and reduces metabolism in the presence of various antimicrobials related to cell-wall synthesis (*i.e*., carbenicillin and cefoxitin) [42]. In the present study, we have characterized a new GhoT-like toxin, OrtT, in *E. coli.* OrtT is a small, hydrophobic peptide that shares 63% protein identity with GhoT, and we have demonstrated that: (i) OrtT strongly inhibits growth and causes cell lysis; (ii) removal of the start codon encoding *ortT* restores growth proving OrtT is active as a protein; (iii) OrtT damages the cell membrane and depletes cellular ATP, (iv) the F38R substitution reduces OrtT toxicity; (v) unlike GhoT, OrtT is most likely an orphan toxin as it is neither encoded by a bicistronic operon nor under *in trans* regulation by an antitoxin encoded elsewhere in the genome; (vi) OrtT reduces cellular metabolism in the presence of antimicrobials like TMP and SMZ; and (vii) *ortT* is activated under stringent conditions; so, its role in cell physiology appears to be to reduce growth during nutritional stress.

Based on the structural homology between GhoT and OrtT, we introduced the same mutation (*i.e*., F38R) in OrtT that allowed us previously to create a non-toxic version of GhoT [42] in order to characterize OrtT. Despite the absence of a hydrophobic interaction between Phe38 and Ile21 (that presumably stabilized GhoT) in OrtT (Figure 2A), toxicity was reduced upon F38R substitution indicating OrtT might be functionally similar to GhoT. Moreover, similarities in the morphological changes in the membrane upon OrtT and GhoT production (visualized via TEM) indicates that these toxins might be utilized in a similar fashion by the cells to escape unfavorable environmental stimuli. Surprisingly, deletion of *ortT* did not have the same impact on metabolism in presence of carbenicillin as *ghoT*. In addition, *ortT* was advantageous for the cells in response to a completely different set of antibiotics, TMP and SMZ, related to THF synthesis whereas the *ghoT* mutation was unrecognizable under the same conditions. Hence, OrtT serves a different physiological role than GhoT.

Furthermore, *ortT* mRNA contains two MqsR-preferred 5'-GCU cleavage sites [56] which are absent in *ghoT* mRNA indicating OrtT is likely not active under conditions that activate MqsR whereas MqsR activates GhoT [32]. Unlike GhoT, OrtT also increases swarming motility [57]. Collectively, our results demonstrate that OrtT is a new toxin despite being a homolog of GhoT. Our findings indicate that cells utilize these two similar toxins for different physiological roles; *i.e.*, they utilize the two toxins for different stress conditions. The reduced growth via OrtT under TMP antimicrobial stress could be an outcome of energy depletion from membrane damage by OrtT just like GhoT [42]; however, closer scrutiny is required to evaluate the molecular mechanism of how these toxins are activated in response to specific environmental cues (*i.e*., reduction in THF level or nutrient limiting condition).

**Figure 6:**
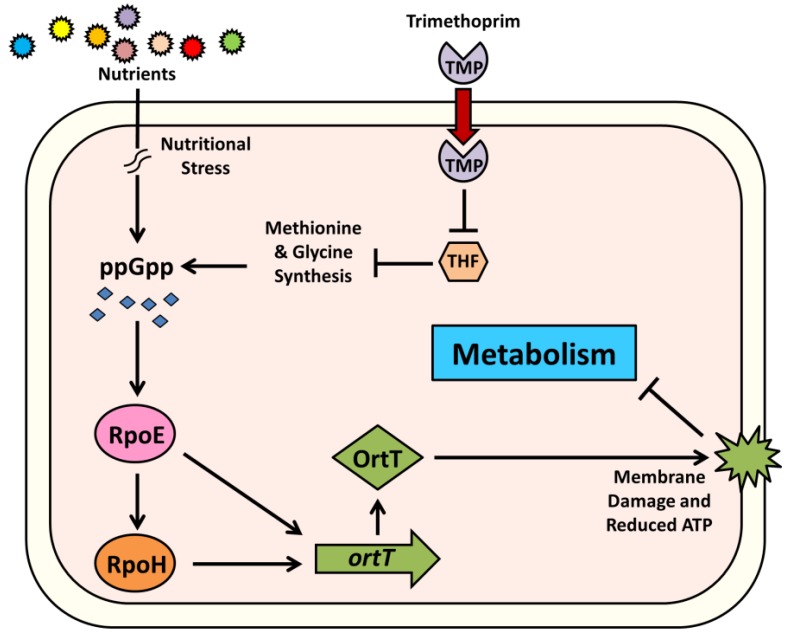
Schematic of proposed mechanism for OrtT-mediated reduction in metabolism under stringent condition. Stringent response or ppGpp accumulation is induced under nutritional stress. TMP treatment also induces ppGpp as reduction in THF level leads to depletion in glycine and methionine. Increased amount of ppGpp then activates the alternative sigma factor RpoE. RpoE in turn activates another stress-related sigma factor RpoH. *ortT* is under RpoE and RpoH regulation and this way, OrtT is activated to damage cellular membrane and to reduce intracellular ATP level. Thus, activation of OrtT leads to reduction in cellular metabolism during stringent response. The symbol → indicates induction and ⊥ indicates repression.

TMP treatment causes immediate accumulation of ppGpp [49] as it reduces THF and induces the stringent response. This nutritional stress response is triggered by the initial rapid depletion of glycine [5]. If glycine is supplied exogenously, the second metabolite pool to be exhausted is ATP [5]. *ortT* is predicted to be under RpoE (σ^E^) and RpoH (σ^H^) regulation, and our results confirm this prediction. RpoE is the stress response sigma factor for extracytoplasmic stress [53]. However, RpoE is also induced by the stringent response alarmone ppGpp [53]. Furthermore, the heat shock sigma factor RpoH is induced by RpoE [54]. This suggests that accumulation of ppGpp due to glycine depletion induces *ortT* expression via activation of RpoH and RpoE. OrtT in turn damages cellular membrane and contributes to reduce the intracellular ATP due to PMF disruption or via direct leakage of intracellular ATP. In this manner, when THF is depleted, the stringent response is activated to shut down growth via accumulation of ppGpp and OrtT (Figure 6). Therefore, OrtT appears to be part of the stringent response, and since it lacks an antitoxin, perhaps OrtT is employed as a “fail-safe” regulator, the activation of which ensures attainment of a non-growing state even faster through exhaustion of the ATP pool.

## 4. Experimental Section

### 4.1. Bacterial Strains, Plasmids, and Growth Conditions

The bacterial strains and plasmids used in this study are listed in Table 2. All experiments were conducted in lysogeny broth (LB) [58] at 37 °C unless stated otherwise. The Keio collection [59] was used to compare isogenic single gene mutations, and the ASKA library [60] was used to produce proteins in *E. coli*. The kanamycin resistance cassette was removed from Δ*ortT* using pCP20 [61] and the *ortT* deletion and kanamycin cassette removal were verified by PCR amplification using the *ortT-*up-F and *ortT*-down-R primers (Table 3) and by sequencing the PCR product using the *ortT-*up-F primer. Cell growth was assayed using the turbidity at 600 nm, chloramphenicol (30 µg/mL) was used to maintain the pCA24N-based plasmids [60], and kanamycin (50 µg/mL) was used to maintain pBS(Kan)-based plasmids [62].

**Table 2:** Bacterial strains and plasmids used in this study. Double colon (::) indicates fusion of a promoter and gene.

Strains	Genotype	Source
BW25113	*rrnB3* Δ*lacZ4787 hsdR514* Δ(*araBAD*)*567* Δ(*rhaBAD*)*568 rph*-*1*	[59]
BW25113 ∆*ortT*	BW25113 Δ*ortT* Ω Km^R^	[59]
BW25113 ∆*ghoT ∆kan*	BW25113 Δ*ghoT*	[42]
BW25113 ∆*ortT ∆kan*	BW25113 Δ*ortT*	This study
BW25113 ∆*mqsR* ∆*mqsA ∆kan*	BW25113 ∆*mqsR* ∆*mqsA*	[43]
**Plasmids**	**Genotype**	**Source**
pCA24N	Cm^R^; *lacI*^q^	[60]
pCA24N-*ghoT*	Cm^R^; *lacI*^q^, P_T5-lac_::*ghoT*^+^	[60]
pCA24N-*ortT*	Cm^R^; *lacI*^q^, P_T5-lac_::*ortT*^+^	[60]
pCA24N-*relA*	Cm^R^; *lacI*^q^, P_T5-lac_::*relA*^+^	[60]
pCA24N-*ortT*ACG	Cm^R^; *lacI*^q^, P*_T5-lac_*::*ortT*^+^ with start codon A*T*G replaced with A*C*G	This study
pCA24N-*ortT*F38R	Cm^R^; *lacI*^q^, P*_T5-lac_*::*ortT*^+^ with TT changed to CG at nucleotide position 160-161 relative to the 6xHis-GhoT start codon	This study
pCA24N-*ghoT*F38R	Cm^R^; *lacI*^q^, pCA24N P*_T5-lac_*::*ghoT*^+^ with TT changed to CG at nucleotide position 160-161 relative to the 6xHis-GhoT start codon	[42]
pCA24N-*ortT*-*ydcY*	Cm^R^; *lacI*^q^, pCA24N P*_T5-lac_*::*ortT*^+^-*ydcY^+^*	This study
pCA24N-*ortT*-*ghoS*	Cm^R^; *lacI*^q^, pCA24N P*_T5-lac_*::*ortT*^+^-*ghoS^+^*	This study
pCA24N-*ortT*-B	Cm^R^; *lacI*^q^, pCA24N P*_T5-lac_*::*ortT*^+^ with the *Bam*HI site in the *N*-terminus linker deleted.	This study
pCP20	Ap^R^; Cm^R^, *FLP*^+^, λ *c*I857^+^, λ *p*_R_Rep^ts^	[61]
pBS(Kan)	Km^R^; cloning vector	[62]
pBS(Kan)-*mqsR*	Km^R^; pBS(Kan) P_lac_::*mqsR*^+^	[43]
pBS(Kan)-*mqsR-mqsA*	Km^R^; pBS(Kan) P_lac_::*mqsR*^+^-*mqsA*^+^	[43]

Cm^R^ and Km^R^ are chloramphenicol and kanamycin resistance, respectively.

**Table 3:** Oligonucleotides used for plasmid construction (synthetic RBS is double underlined), site directed mutagenesis (target mutated nucleotides are underlined), verification of kanamycin cassette removal, and qRT-PCR.

Purpose/Name	Sequence (5' to 3')
**Plasmid construction**	
*ydcY*-*Sal*I-F	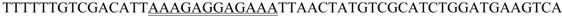
*ydcY*-*Hin*dIII-R	TTTTTTGTCGACAAGCTTAGACGCTCATTTTAATCAGAGGATGGTG
*ghoS*-*Sal*I-F	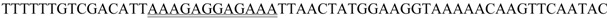
*ghoS*-*Hin*dIII-R	TTTTTTGTCGACAAGCTTCTTATCCTTCCTGGCTACTTGTAAAACTGAC
UTR-F	TTTTTTGTCGACACAGTTCTACTGGAAACATTCATTTTTGC
UTR-R	TTTTTTAAGCTTCAATTTGTGGCGCAATTTTACTTGTG
**Site-directed mutagenesis**	
*ghoT*-ACG-F	AAAGAGGAGAAATTAACTACGAGAGGATCTCACCAT
*ghoT*-ACG-R	ATGGTGAGATCCTCTCGTAGTTAATTTCTCCTCTTT
*ortT*F38R-F	TCCGTTTCTTAAGCGCCCGTCTGGTGGGGGCAAC
*ortT*F38R-R	ATGTTGCCCCCACCAGACGGGCGCTTAAGAAACGGA
*ortT*-ACG-Chrom-F	GACGCCCGTACACGTCTCTCTATCAACA
*ortT*-ACG-Chrom-R	TGTTGATAGAGAGACGTGTACGGGCGTC
*Bam*HI-F	GAGGATCTCACCATCACCATCACCATACGGCCCTGAGGGCCTCTCTCTATCAACAC
*Bam*HI-R	GTGTTGATAGAGAGAGGCCCTCAGGGCCGTATGGTGATGGTGATGGTGAGATCCTC
**Verification of *Kan* insertion/removal**	
*ortT-*up-F	ATGGATAAGGGCAAGTTGCTGTTTGATG
*ortT*-down-R	CCAATTTGTGGCGCAATTTTACTTGTG
**qRT-PCR**	
*gyrA*-F	GTCATGCCAACCAAAATTCCTAAC
*gyrA*-R	TCATCATCAATATACGCCAGACAAC
*ortT*-F	AATCGCATTTCTTATCACCTGG
*ortT*-R	GAAACTCATCGGCCATGTTG

F indicates forward primer and R indicates reverse primer.

### 4.2. Construction of pCA24N-ortT-ydcY, pCA24N-ortT-ghoS, and pCA24N-ortT-UTR

The putative antitoxin genes, *ydcY* and *ghoS* were PCR-amplified from *E. coli* K12 BW25113 chromosomal DNA using primer pair *ydcY*-*Sal*I-F/*ydcY*-*Hin*dIII-R and *ghoS*-*Sal*I-F/*ghoS*-*Hin*dIII-R respectively. The forward primers were designed to include the synthetic RBS from pCA24N plasmid [60] (Table 3). The pCA24N-*ortT*-UTR plasmid was constructed by cloning the intervening DNA flanking *ortT* from the BW25113 chromosome into pCA24N-*ortT*. To do so, the 217 bp upstream intergenic region (between *yncL* and *ortT*), an inactivated *ortT* (174 bp), and the 381 bp downstream intergenic region including *ydcY* (a total of 772 bp, Figure S2D) was PCR amplified using UTR-F/R primer pair (Table 3). The chromosomal *ortT* gene was inactivated by changing the start codon (*i.e*., A*T*G→A*C*G) via site-directed mutagenesis using the primer pair *ortT*-ACG-chrom-F/R (Table 3) so that the final plasmid (*i.e*., pCA24N-*ortT*-UTR) that was used in the growth experiment (Figure 3B) had only one copy of active *ortT* like the other two plasmids (*i.e*., pCA24N-*ortT*-*ydcY* and pCA24N-*ortT*-*ghoS*). PCR products were purified using a Promega PCR product purification kit (Promega, Madison, WI, USA). The purified PCR product and the vector pCA24N-*ortT* were digested with *Sal*I and *Hin*dIII restriction enzymes (New England Biolabs, Beverly, MA, USA) and then ligated using T4 DNA ligase. The ligation mixture was transformed into BW25113 competent cells and the resulted plasmids were verified by sequencing.

### 4.3. Genomic Library Construction

The genome library was constructed by using *Bam*HI (to cut the vector) and *Sau*3AI (to cut the genomic DNA) restriction enzymes (New England Biolabs, Beverly, MA, USA) that produce compatible overhangs. Since there are two *Bam*HI sites in the ASKA plasmid pCA24N-*ortT* (*i.e*., one in the *N*-terminus linker of *ortT* and the second downstream of *ortT*), the vector was modified by deleting the first *Bam*HI site in the pCA24N-*ortT* plasmid via site-directed mutagenesis with the *Bam*HI-F/R primer pair (Table 3). The resulting plasmid, pCA24N-*ortT*-B (with a single *Bam*HI site) was digested with *Bam*HI and dephosphorylated with rSAP phosphatase (New England Biolabs, Beverly, MA, USA) to prevent self-ligation. BW25113 genomic DNA was partially digested with *Sau*3AI and ~2–4 Kb genome fragments were gel-purified and ligated into the linearized vector using T4 DNA ligase. The ligation mixture was transformed into BW25113 competent cells. The library construction was verified by sequencing three random clones (that were not challenged with IPTG), which had inserts mapping to different places in the genome (*i.e*., fragments including *bepA*, *yiaU*, and *elfG*). The average insert size of the library was 622 bp found by digesting the plasmids obtained from 25 random clones with SapI (cuts near the origin of the plasmids). The library was then screened by growing the clones with 1 mM IPTG to induce *ortT* expression. Surviving clones (~0.25% of the library) that grew in presence of IPTG were analyzed by digesting their plasmids with *Afl*II which cuts within the *ortT* coding region; none of the plasmids were cut by *Afl*II whereas the plasmids could be digested with *Sap*I. Moreover, the linearized plasmids were shorter compared to the plasmid that contains *ortT* (pCA24N-*ortT*-B) which indicates the toxin gene was missing in the survivor plasmids. Approximately, 21,314 clones were screened in this experiment; hence, the genome coverage was 2.9 times (*i.e*., 21,314 clones × 622 bp/4.6 Mbp).

### 4.4. Site-Directed Mutagenesis

Site-directed mutagenesis PCR was performed using pCA24N-*ortT* (Table 2) as the template with *ghoT*-ACG-f/*ghoT*-ACG-r for removing the start codon and with *ortT*F38R-f/*ortT*F38R-r for changing Phe38 to Arg (Table 3). The PCR products were recovered subjected to *Dpn*I digestion for removing the template plasmids and the digestion product were then transformed into *E. coli* BW25113 wild-type competent cells. The plasmids were isolated from resulted transformants and the desired mutations were confirmed by sequencing check (Table 2).

### 4.5. Toxicity Assay

For the toxicity assays, overnight cultures were refreshed in fresh LB medium with chloramphenicol to an initial turbidity at 600 nm of 0.05, cells were allowed to grow to turbidity of 0.1, induced with 1 mM IPTG (for OrtT is a proteic toxin and OrtT is an orphan toxin) or 0.1 mM IPTG (for growth assays with F38R mutants) and turbidity at 600 nm was recorded every hour for monitoring cell growth. For performing streak toxicity tests, overnight cultures were streaked on LB plates supplemented with chloramphenicol and with or without IPTG (same as liquid growth test) and incubated overnight.

### 4.6. Lysis Assay

To determine whether OrtT overexpression causes cell lysis, overnight cultures were refreshed in fresh LB medium with chloramphenicol to an initial turbidity at 600 nm of 0.05, cells were grown to turbidity of 0.5, then induced with 2 mM IPTG (time zero). Cell sample was collected every 30 min by harvesting 1 mL culture for assessing cell viability. Harvested cell pellets were washed and diluted by 10^2^ to 10^7^ via 10-fold serial dilution steps in 0.85% NaCl solution and 10 µL of diluted cell suspension was plated on LB agar plate with chloramphenicol for enumerating colony forming unit (CFU) per mL [63].

### 4.7. TEM

TEM was performed for overexpressing OrtT as described previously [42]. For preparing cell samples for TEM, overnight cultures of BW25113/pCA24N-*ortT* and BW25113/pCA24N-(-*gfp*) were refreshed to a turbidity at 600 nm of 0.05, grown in LB-chloramphenicol to a turbidity at 600 nm of 0.2, and then 1 mM of IPTG was added to induce OrtT production for 4 h. Cells were harvested and fixed with primary fixative (1.5% paraformaldehyde and 2.5% glutaraldehyde, and 4% sucrose in 100 mM of sodium cacodylate buffer, pH 7.4) at room temperature for 15 min, then 4 °C for 12 h. Cell pellets were then washed three times with buffer (0.1 M sodium cacodylate buffer, pH 7.4) for 5 min. Secondary fixation was carried by fixing the cell pellets with 1% osmium tetroxide in buffer for 1 h in dark, followed by three washes with buffer and one wash with sterile MilliQ water. Cells were then subjected to en bloc staining by treating with 2% uranyl acetate in the dark for 1 h. Next, dehydration step was performed by applying a graded ethanol series (50%, 70%, 85%, 95% and three times with 100% ethanol, for 5 min each), and 100% acetone (three times, 5 min each). The dehydrated cells were then embedded into epoxy resins after three epoxy exchange for at least 12 h at 60 °C. The hardened cell samples were then sectioned into thin specimens (70 nm thick) using an ultramicrotome (UC6, Leica, Buffalo Grove, IL, USA). Positive staining was performed by staining the specimens with uranyl acetate for 6 min and lead citrate for another 6 min. The stained specimens were then examined on a FEI Tecnai G2 Spirit BioTwin TEM (Penn State Microscopy and Cytometry Facility, University Park, PA, USA) at an accelerating voltage of 120 kV.

### 4.8. ATP Assay

The ATP assay was conducted as described previously [42] using the luciferase kit (ENLITEN ATP assay, Promega, Madison, WI, USA). The RLU values were normalized for growth by the optical density (600 nm) at the time of harvest for each sample and were converted to relative numbers using the values of the strain with the empty plasmid.

### 4.9. Persister Assay

The persister assay was conducted as described [27]. Briefly, overnight cultures were diluted to an initial turbidity at 600 nm of 0.05 and grown with chloramphenicol (30 µg/mL) to a turbidity of 1.0, then 1 mM IPTG was used to induce OrtT. After 2 h of induction, cells were washed, adjusted to a turbidity of 1.0 with chloramphenicol (30 µg/mL), and were treated with ampicillin (100 µg/mL) or ciprofloxacin (5 µg/mL) for another 3 h. Cell viability was determined for each sample before and after ampicillin treatment by applying 10 µL drops in serial dilutions [63] plates with chloramphenicol. Two independent cultures per strain were evaluated.

### 4.10. Metabolism and Relevant Growth Assay

Metabolic activities in response to antimicrobials were assayed using reagents from Biolog, Inc. (Hayward, CA, USA) following BioLog’s procedure [64]. Briefly, cells were grown to turbidity at 600 nm of 1.0, diluting to a turbidity of 0.07 in IF-10a (Cat. No. 72264), and then further diluted 200 fold into a reagent mixture containing IF-10a, BioLog Redox Dye D (Cat. No. 74224) and a rich medium (2.0 g of tryptone, 1.0 g of yeast extract, and 1.0 g of NaCl per liter) to a final turbidity of 0.00035. Next, 90 µL volume of this diluted cell suspension was transferred into 96-well microtiter plates prepared with 10 µL of 10× antibiotics (*i.e.*, carbenicillin, TMP, and SMZ), or solvent only (water or DMSO). The metabolic activity (NADH production) was monitored by taking hourly measurement of the absorbance at 590 nm which indicates the intracellular reducing state based on the generation of formazane (purple) from the tetrazolium dye. For performing the growth assay under these conditions, the BioLog Redox Dye was replaced with water and growth was monitored by measuring the absorbance at 600 nm.

### 4.11. qRT-PCR

Total RNA was isolated as described previously [65] using Qiagen RNeasy mini kit (Valencia, CA, USA). Overnight cultures were diluted in 25 mL M9 medium [58] supplemented with 0.4% glucose to an initial turbidity at 600 nm of 0.05. For stringent conditions, the inducer (*i.e.*, 75 µg/mL TMP, 100 µg/mL MUP, 1 mg/mL SHX or 1 mM IPTG for *relA* induction) was added when the cells reached the exponential phase (turbidity at 600 nm of 0.2~0.4), and the cells were induced for 2 h before isolating RNA. For TMP, MUP, and SHX treatment, BW25113 was used. For RelA production, BW25113 carrying pCA24N-*relA* (Table 2) or empty plasmid pCA24N (negative control) were used. For the heat-shock stress experiment, overnight cultures of BW25113 were diluted in M9 medium supplemented with 0.4% glucose and 0.4% casamino acid, and cells were then incubated at 30 °C. In the exponential phase (turbidity at 600 nm of 0.3), 10 mL cell samples were harvested for the no stress control, and the rest of the cells were heat shocked by incubating at 43 °C for 30 min. qRT-PCR was performed according to the manufacturer’s instructions using the *Power* SYBR Green RNA-to-*C*_T_ *1-Step* kit (Life Technologies, Carlsbad, CA, USA) using 100 ng of total RNA as the template. Primers (Table 3) were annealed at 60 °C, and fold changes were calculated using 2^−∆∆CT^ formula [66] with *gyrA* as the internal control for normalizing all data.

### 4.12. Additional Methods

Conditions for tricine-SDS-PAGE and the Western blot analysis are described in the Supplementary Methods.

## 5. Conclusions

Although previous studies have predicted independent occurrences of toxins [67,68,69], demonstration of an active orphan toxin has not been achieved previously. Our results show that there is a possibility that a toxin can exist solely by itself, and it does so with a distinct biological role related to the stringent response. These findings raise an intriguing question as to why the cells have a toxin in the chromosome that is not “guarded” by an antitoxin. It appears that instead of an antitoxin, another strategy is that cells may regulate toxins via tight regulation such as the two tandem promoters regulated by alternative sigma factors as demonstrated for *ortT*. Further research is required to determine how OrtT is inactivated after stress; however, our results create a new simpler paradigm for reducing cellular metabolism during stress using an orphan toxin.

## Supplementary Materials

Supplementary materials can be accessed at: http://www.mdpi.com/2072-6651/7/2/0299/s1.
